# Risk of new-onset diabetes among patients treated with statins according to hypertension and gender: Results from a nationwide health-screening cohort

**DOI:** 10.1371/journal.pone.0195459

**Published:** 2018-04-09

**Authors:** Sang-Eun Lee, Ji Min Sung, In-Jeong Cho, Hyeon Chang Kim, Hyuk-Jae Chang

**Affiliations:** 1 Division of Cardiology, Department of Internal Medicine, Severance Cardiovascular Hospital, Yonsei University Health System, Seoul, Republic of Korea; 2 Integrative Cardiovascular Imaging Research Center, Yonsei University College of Medicine, Yonsei University Health System, Seoul, Republic of Korea; 3 Department of Preventive Medicine, Yonsei University College of Medicine, Yonsei University Health System, Seoul, Republic of Korea; Universita degli Studi di Ferrara, ITALY

## Abstract

**Background:**

Statins have been known to increase the risk of incident type 2 diabetes mellitus (DM); however, other factors, especially hypertension, are also associated with DM development.

**Objective:**

We investigated whether statin use increases the risk of DM and further analyzed whether the relation between statin use and incident DM differs according to the presence of hypertension and gender.

**Methods:**

From a nationwide health-screening cohort, 40,164 participants with total cholesterol levels ≥eve mg/dL and without pre-diagnosed DM, cardiovascular disease, or cancer, who underwent a series of regular health check-ups, were enrolled. Statin users were defined as participants who were prescribed statins more than twice during 6 months.

**Results:**

There were 17,798 statin non-users and 22,366 statin users. During 7.66±3.21 years of follow-up, incident DM developed in 5.68% of statin non-users and 7.64% of statin users. Among the entire study population, statin use was associated with new-onset DM after adjusting for clinical risk factors. In sub-analysis according to hypertension, statin use significantly increased the risk of incident DM only in normotensive patients [hazard ratio (HR) 1.31, 95% confidence interval (CI) 1.09 to 1.58, *p* = 0.004], and not in hypertensive patients (*p*>0.05). Furthermore, continuous statin use was strongly associated with new-onset DM in women, regardless of hypertension presence (all *p*<0.05). However, in men, statin was associated with new-onset DM only in normotensive males (HR 1.61, 95% CI 1.35 to 1.92, *p*<0.001) and not in hypertensive males (*p*>0.05).

**Conclusions:**

Statin use increased the risk of new-onset DM only in normotensive patients and hypertensive women, suggesting that these groups should be more carefully monitored for the development of DM during the course of follow-up.

## Introduction

The impact of statins on the development of type 2 diabetes mellitus (DM) has been studied in both randomized controlled trials and meta-analyses [1–5]. Based on the results, the risk of new-onset DM has been added to labels of statins by the US Food and Drug Administration [6]. However, because DM itself is often associated with dyslipidemia, hypertension, obesity, and elevated glucose levels, it remains controversial whether only patients who are already at high risk for developing DM are susceptible to statin-induced DM or patients who are at low risk for type 2 DM also have a risk for statin-induced DM. Therefore, all these confounding factors should be considered to determine whether a sex difference exists in susceptibility to the development of new-onset DM associated with statin use.

Especially, it has been shown that hypertensive patients without DM have an increased risk of developing diabetes and a high prevalence of insulin resistance, when compared with the risk and prevalence in normotensive subjects [7,8]. Furthermore, antihypertensive therapy, including both beta-blockers and diuretics, has been shown to increase the risk of new-onset DM [9,10]. As a result, recent guidelines do not recommend thiazides or beta-blockers in patients with hypertension who are at high risk of developing diabetes [11]. Therefore, it is required to consider the presence of hypertension separately when assessing the impact of statins on the development of type 2 DM.

Therefore, in the present study, we first investigated whether a relation exists between statin use and incident type 2 DM in a large cohort of a relatively healthy population undergoing national health screening. Furthermore, we investigated whether the effect of statins on incident DM is dependent on the presence of hypertension and evaluated sex differences.

## Materials and methods

### Study population

In the Republic of Korea, the National Health Insurance System (NHIS) is compulsory for all citizens, and it is important in the management and maintenance of health service use. The NHIS operates a biennial health-screening program at the national level, including a general health examination for all its participants over the age of 40 years.

In 2016, data from the NHIS-National Health-screening Cohort (NHIS-HEALS), representing approximately 10% of the total source population, were released. The cohort consisted of 514,795 randomly sampled individuals who had started their biennial NHIS health screening between years 2002 and 2003. Each individual in the cohort had received follow-up until either the end of study period (2013) or the time of health service disqualification due to death or emigration, and had their health screening every two years. The NHIS-HEALS contains eligibility and demographic information regarding health insurance and medical aid beneficiaries, histories of medical treatment or disease, details of medical bills, and drug prescriptions.

From the entire database, statin users were identified as patients who were prescribed statins more than twice during 6 months (n = 137,784), and statin nonusers were identified as patients who were not prescribed statins between 2002 and 2013 (n = 330,805). Among these 468,589 patients, we excluded those who met the following criteria: 1) treatment with statins before the first examination (n = 39,039), 2) treatment with statins after the diagnosis of DM (n = 17,978), 3) only one examination (n = 53,786), 4) an elevated fasting glucose level at the baseline examination (n = 13,564), 5) a low total cholesterol level (<240 mg/dL) at the baseline examination (n = 297,815), 6) previous history of DM (10th revision of the International Classification of Diseases [ICD-10] code E11 with prescription history of diabetic drugs), cardiac disease (ICD-10 code I21, I22, or I23), stroke (ICD-10 code I63), malignancy (ICD-10 codes C00–C97) (n = 4,875), and 7) previous history of impaired fasting glucose or gestational DM (ICD-10 codes E10–14, O24, or R73) (n = 1,367). The final dataset included 40,164 patients (17,798 statin non-user and 22,366 statin users). The date of enrollment was set as the date on which statin was first prescribed.

This study was approved by the institutional review board of Yonsei University College of Medicine. Informed consent was not obtained from each participant owing to the characteristics of the data obtained from the NHIS and the fact that the data were fully de-identified and anonymized.

### Clinical variables

Laboratory tests, including fasting glucose and total cholesterol tests, were performed after overnight fasting at each examination visit. Systolic and diastolic blood pressure, body weight, height, and waist circumference were measured at every visit. Body mass index (BMI) was calculated using the documented body weight and height. Detailed histories of alcohol consumption and smoking status were obtained via a questionnaire.

The medical history of the participants was assessed using a combination of the following: clinical and pharmaceutical codes of ICD-10, list of previously prescribed medicines, and past medical information. Prescription of well-known diabetogenic drugs other than statins (beta-blockers and hydrochlorothiazide) was identified and adjusted [9,10,12].

### Study endpoint

The primary endpoint was the development of new-onset DM, identified according to the newly entered ICD-10 codes E11–14, with prescription of diabetic drugs.

### Statistical analysis

Variables are reported as percentages or means ± standard deviations (SDs) for normally distributed variables, and medians (with 25–75% ranges) for non-normally distributed variables, as necessary. The chi-square test was used for nominal variables. Development rate of new-onset DM was computed by time-dependent Cox regression. Hazard ratios (HR) were calculated to model the effects of statins on development of DM in univariate and multivariate analyses. Variables in multivariate model were age, sex, body mass index, blood pressure, fasting blood glucose level, total cholesterol level, history of smoking and drinking, exercise, family history of type 2 DM, duration of statin treatment, and history of taking beta-blocker or hydrochlorothiazide. A *p*-value <0.05 was considered statistically significant. All analyses were performed using SAS, version 9.4 (SAS Institute Inc., Cary, NC, USA).

## Results

### Clinical and laboratory results at baseline and last follow-up

Overall, 40,164 patients (mean age, 55.1±9.0 years; 47.0% male) were enrolled from the NHIS-HEALS. The mean total follow-up duration was 7.7±3.2 years (Table 1). Among the entire study population, 12,527 (31.2%) patients were diagnosed with hypertension at baseline. There were 17,798 statin non-users (44.3%; mean age, 52.2±9.4 years; 59.9% male) and 22,366 statin users (55.7%; mean age, 57.4±8.0 years; 36.8% male). At the time of enrollment, the mean total cholesterol level of the entire study population was 264.8±25.9 mg/dL and the mean fasting blood glucose level was 94.0±12.5 mg/dL.

**Table 1:** Baseline clinical characteristics and laboratory results.

Risk factors	Total (n = 40,164)	Statin (-) (n = 17,798)	Statin (+) (n = 22,366)
Age (years)	55.1±9.0	52.2±9.4	57.4±8.0
Male gender, n (%)	18,882 (47.0)	10,653 (59.9)	8,229 (36.8)
Follow-up duration (years)	7.7±3.2	10.2±1.7	5.6±2.6
BMI (kg/m^2^)	24.5±2.8	24.4±2.8	24.6±2.8
SBP (mmHg)	128.6±17.4	127.4±17.6	129.5±17.1
DBP (mmHg)	80.5±11.3	80.4±11.6	80.5±11.0
Total Cholesterol (mg/dL)	264.8±25.9	258.7±23.4	269.7±26.8
FSG (mg/dL)	94.0±12.5	92.8±13.1	95.0±11.9
Smoking, n (%)	7,735 (19.3)	4,916 (27.6)	2,819 (12.6)
Alcohol use, n (%)	15,822 (39.4)	8,432 (47.4)	7,390 (33.0)
Exercise, n (%)	21,148 (52.7)	7,694 (43.2)	13,454 (60.2)
FHx of type 2 DM, n (%)	5,504 (13.7)	2,541 (14.3)	2,963 (13.3)
Duration of statin (years)	-	-	4.4±2.9
Drug history, n (%)	6,057 (15.1)	819 (4.6)	5,238 (23.4)
Use of beta-blocker	2,181 (5.4)	359 (2.0)	1822 (8.2)
Use of thiazide	3,876 (9.7)	460 (2.6)	3,416 (15.3)

BMI, body mass index; DBP, diastolic blood pressure; DM, diabetes mellitus; FHx, family history; FSG, fasting serum glucose; SBP, systolic blood pressure

At follow up, the total cholesterol level decreased when compared to the level at baseline, regardless of statin use, although the absolute difference was greater in statin users (Δ56.0 mg/dL) than in non-users (Δ32.3 mg/dL) (S1 Table). The decreases in the total cholesterol level among both statin users and non-users were maintained when the population was divided according to the presence of hypertension. In contrast to the total cholesterol level, the fasting serum glucose level increased in both statin users and non-users.

### Time-dependent multivariate Cox regression analysis for development of DM

Development of incident type 2 DM was noted in 5.7% of statin non-users (1,011 patients) and 7.6% of statin users (1,709 patients) (*p* = 0.045). When considering the entire study population (Table 2), statin use was associated with the development of new-onset type 2 DM (hazard ratio [HR]: 1.66, 95% confidence interval [CI]: 1.49 to 1.85, *p*<0.001) after adjusting for age, sex, BMI, blood pressure, fasting glucose level, total cholesterol level, smoking, drinking, exercise, family history of type 2 DM, and history of taking a beta-blocker or hydrochlorothiazide, using time-dependent Cox regression analysis.

**Table 2:** Time dependent multivariate Cox regression analysis for development of new-onset type 2 diabetes mellitus*.

Risk factors	Total patients		HTN (-)		HTN (+)	
	(n = 40,164)		(n = 27,637)		(n = 12,827)	
	HR (95% CI)	*P*	HR (95% CI)	*P*	HR (95% CI)	*P*
Statin use	1.66 (1.49, 1.85)	< .001	1.31 (1.09, 1.58)	0.004	1.18 (0.96, 1.46)	0.114
Female	1.24 (1.09, 1.41)	0.001	1.29 (1.03, 1.60)	0.024	1.10 (0.90, 1.33)	0.361
Age	1.02 (1.01, 1.03)	< .001	1.01 (0.99, 1.03)	0.217	1.02 (1.00, 1.03)	0.021
BMI	1.08 (1.07, 1.10)	< .001	1.08 (1.06, 1.11)	< .001	1.06 (1.04, 1.09)	< .001
SBP	1.01 (1.00, 1.01)	0.004	1.01 (1.00, 1.02)	0.051	0.10 (0.99, 1.01)	0.640
DBP	1.00 (0.99, 1.01)	0.672	1.00 (0.99, 1.01)	0.729	1.00 (0.99, 1.01)	0.428
FSG	1.04 (1.04, 1.04)	< .001	1.04 (1.04, 1.04)	< .001	1.04 (1.03, 1.04)	< .001
TC	0.99 (0.99, 1.00)	< .001	1.00 (0.99, 1.00)	< .001	1.00 (0.99, 1.00)	< .001
Smoking	1.37 (1.16, 1.61)	< .001	1.26 (0.95, 1.66)	0.106	1.32 (1.03, 1.70)	0.028
Drink	0.73 (0.64, 0.82)	< .001	0.88 (0.70, 1.10)	0.247	0.76 (0.62, 0.94)	0.009
Exercise	0.89 (0.81, 0.99)	0.02	0.87 (0.73, 1.04)	0.119	0.88 (0.75, 1.03)	0.099
Family DM	1.34 (1.18, 1.52)	< .001	1.49 (1.18, 1.89)	0.001	1.28 (1.04, 1.59)	0.022
Drug	1.04 (0.92, 1.17)	0.526	0.95 (0.70, 1.31)	0.773	0.93 (0.77, 1.09)	0.362

BMI, body mass index; CI, confidence interval; DBP, diastolic blood pressure; DM, diabetes mellitus; FHx, family history; FSG, fasting serum glucose; HR, hazard ratio; HTN, hypertension; SBP, systolic blood pressure; TC, total cholesterol

*Adjusted with age, sex, body mass index, blood pressure, fasting serum glucose, smoking, drinking, exercise, family history of type 2 DM, total cholesterol level and history of taking beta-blocker or hydrochlorothiazide

To determine the impact of hypertension on the development of DM associated with statin use, patients were divided into normotensive and hypertensive groups. Statins tended to be more commonly prescribed in hypertensive patients than in normotensive patients (84.3% vs. 42.7%, *p*<0.001). Statin therapy significantly increased the risk of incident type 2 DM only in normotensive patients (HR: 1.31, 95% CI: 1.09 to 1.58, *p* = 0.0039) and not in hypertensive patients (HR: 1.18, 95% CI: 0.96 to 1.46, *p* = 0.114). Notably, female gender was a risk factor for development of DM only in normotensive group.

### Sex differences in the impact of hypertension on the development of incident DM with statin therapy

**Fig 1:**
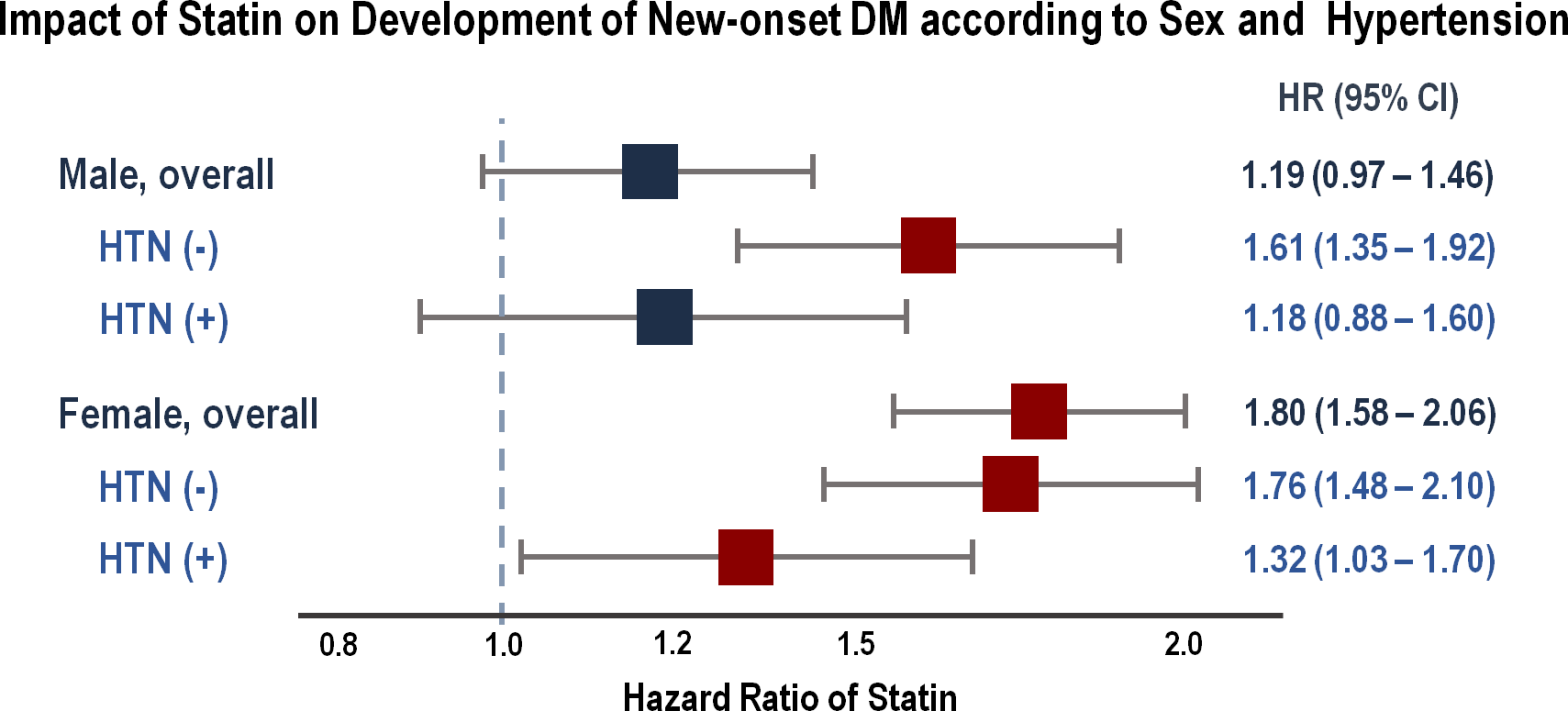
Effect of statins on development of diabetes mellitus according to sex and hypertension. CI, confidence interval; HR, hazard ratio; HTN, hypertension^. *^Adjusted with age, body mass index, blood pressure, fasting serum glucose, smoking, drinking, exercise, family history of type 2 DM, total cholesterol level and history of taking beta-blocker or hydrochlorothiazide.

In the sub-analysis according to sex, in men, statin use was not associated with new-onset DM (HR: 1.19, 95% CI: 0.97 to 1.46, *p* = 0.101), even after correcting for the change in the total cholesterol level (Fig 1). In contrast, in women, statin use was significantly associated with the development of incident DM (HR: 1.80, 95% CI: 1.58 to 2.06, *p*<0.001).

On further dividing the patients according to the presence of hypertension, in men, statin use was not associated with DM among hypertensive patients (HR: 1.18, 95% CI: 0.88 to 1.60, *p* = 0.274) (Fig 1, S2 Table). However, notably, statin use significantly increased the risk of developing DM among normotensive men (HR: 1.61, 95% CI: 1.35 to 1.92, *p*<0.001). In contrast, the presence of hypertension did not influence the impact of statins on the development of DM in women. Statin use was associated with an increased risk of new-onset DM in both normotensive (HR: 1.76, 95% CI: 1.48 to 2.10, *p*<0.001) and hypertensive (HR: 1.32, 95% CI: 1.21 to 1.70, *p* = 0.030) women.

## Discussion

The main findings of the current study using a nationwide time-series health-screening cohort are as follows: (1) statin therapy increased the risk of new-onset type 2 DM at the total population level, and (2) the risk of DM associated with statin use increased only in normotensive patients. Moreover, statin therapy did not show any impact on the development of DM in only hypertensive male patients.

These results are partly consistent with the findings of previous studies that demonstrated an increased risk of new-onset type 2 DM associated with statin use [1–5]. Because type 2 DM is a complex disease and multiple risk factors, including metabolic syndrome, are strongly involved in its development [13], the true pure effect of statins is difficult to acknowledge, as these diseases are often cluster together as metabolic syndrome [14]. Statin users who develop incident DM often have evidence of impaired fasting glucose levels or metabolic syndrome even before starting statin therapy [15,16]. In fact, preexisting risk factors, including baseline fasting serum glucose levels, BMI, fasting triglyceride levels, and hypertension, independently predicted new-onset DM in 3 large randomized trials involving atorvastatin [16]. The fact that statins increased the risk of DM only in normotensive patients in this study supports these previous findings, because in hypertensive patients, multiple clustering factors, other than statin use, were found to be associated with the development of DM.

Moreover, when we further divided the patients according to sex, only normotensive, not hypertensive, patients were prone to acquire DM among men. Previous studies concluded that statin use is a stronger risk factor for the development of type 2 DM in women than men [17,18]. However, it is still unclear whether these reported sex differences in the risk of DM are real or attributable to differences between women and men taking statins with respect to other risk factors for type 2 DM. The present finding of a sex difference in incident DM underscores the importance of setting treatment and monitoring strategies in women and men separately.

Despite the large amount of evidence for a higher incidence of DM with statin use, the underlying mechanisms are not yet well characterized. So far, several potential mechanisms have been suggested. Statins may directly decrease the synthesis of insulin or reduce insulin secretion [19], exacerbating insulin resistance, and/or modify insulin signaling in peripheral target tissues [20]. Another suggested mechanism states that the effect of statins associated with the reduction in cholesterol levels induces DM and not the drug itself [21]. However, in the current study, statin use was independently associated with the risk of DM even after adjusting for the change in the total cholesterol level, which suggests that the drug itself has an influence on the development of DM.

The prevalence of type 2 DM in South Korea drastically increased from 1970 to 2000, and remained relatively stable after 2000. South Korea also ranked 21^st^ among the 30 Organization for Economic Co-operation and Development member countries in terms of adult (aged 20 to 79) diabetes prevalence, according to the International Diabetes Federation report [22,23]. This was accompanied by the increase in socioeconomic status and prevalence of adult obesity [23,24]. However, as a country with one of the highest growth rates, South Korea is expected to have the highest level of prevalence for type 2 DM by the year 2030 [23]. Furthermore, a recent study showed that the prevalence of cardiovascular disease was more than two times higher in diabetic population compared to the general Korean population [23]. These results warrant a need for planning national public health strategies, as well as identification of every possible factor that could be related to development of type 2 DM.

Furthermore, it is also important to identify patients who are at higher risk for type 2 DM in order to guide individual treatment in clinical practice, as recent modifications in the guidelines regarding management of dyslipidemia could increase the global use of statins [25]. Moreover, despite the association between statins and new-onset DM, studies investigating this relationship have equally confirmed the prevailing cardiovascular therapeutic benefit of statins in both men and women [9,15,18,26,27]. The current study found that women and normotensive men were at high risk for developing incident DM and might require more thorough monitoring during the course of follow-up.

The present study has some limitations. First, this study was conducted using observational data of South Korean population, and study subjects are East Asians. Hence, application of the data into clinical practice warrants caution, as results might differ in patients of other ethnicities. Furthermore, due to the nature of observational data, impact of the drug is inevitably susceptible to confounding factors, including indications and clinical backgrounds, which can obscure the true effect of the drug or even imply false associations between the drug and outcomes. However, it should be also acknowledged that, to date, no trials regarding statins were designed to test the effect of statins on new-onset DM, and as a result, trials using a range of methods were collected for meta-analyses. Third, potential selection bias is another inherent limitation of this study. The NHIS only recommends, but does not necessarily obligate, all health insurance subscribers to take regular health examinations. Therefore, the current cohort likely consisted of relatively healthier individuals without very serious diseases or individuals who were concerned about health. However, the data reflects a representative sample of subjects who were prospectively followed. Finally, as glycated hemoglobin was not included in the screening parameters, diagnosis of type 2 DM was made based on ICD-10 codes. However, such disease definition was frequently used in population-based studies with limited variables [22,28], while fasting glucose level and prescription history of diabetic drugs were also considered in defining patients with diabetes.

In conclusion, in this analysis based on a nationwide cohort, statin use was associated with an increased risk of incident DM only in normotensive patients, and only women and normotensive men were susceptible to the development of new-onset DM on statin use. The current study suggests that these patient groups should be more carefully monitored for the development of DM when taking statins.

## Supporting information

S1 Table Clinical and laboratory results at baseline and follow-up. (DOCX)

S2 Table Multivariate Cox regression analysis for development of new-onset type 2 diabetes mellitus according to sex and hypertension. (DOCX)
