# Supplementary material for: Risk of new-onset diabetes among patients treated with statins according to hypertension and gender: Results from a nationwide health-screening cohort
Source: PLoS One. 2018 Apr 9;13(4):e0195459. doi: 10.1371/journal.pone.0195459 (PMC5891021; doi:10.1371/journal.pone.0195459)
Supplement: S1 Table — (DOCX) [file pone.0195459.s001.docx]

**S1 Table. Clinical and laboratory results at baseline and follow-up**

1. **Overall (n=40,164)**

| **Risk factors** | **Statin (-)** | | | **Statin (+)** | | |
| --- | --- | --- | --- | --- | --- | --- |
|  | **(n=17,798)** | | | **(n=22,366)** | | |
|  | **Baseline** | **Follow-up** | **P value** | **Baseline** | **Follow-up** | **P value** |
| **Age (years)** | 52.2±9.4 | 60.9±9.1 | <.001 | 57.43±7.96 | 62.0±8.0 | <.001 |
| **BMI (kg/m^2^)** | 24.4±2.8 | 24.1±2.9 | <.001 | 24.62±2.81 | 24.6±2.9 | 0.007 |
| **SBP (mmHg)** | 127.4±17.6 | 126.7±15.7 | <.001 | 129.53±17.13 | 126.4±14.7 | <.001 |
| **DBP (mmHg)** | 80.4±11.6 | 78.4±10.2 | <.001 | 80.46±11.03 | 77.5±9.4 | <.001 |
| **Total cholesterol (mg/dL)** | 258.7±23.4 | 226.4±34.8 | <.001 | 269.67±26.81 | 213.7±45.8 | <.001 |
| **FSG (mg/dL)** | 92.8±13.1 | 99.8±21.6 | <.001 | 94.98±11.87 | 99.4±18.7 | <.001 |
| **Smoking, n (%)** | 4,916 (27.6) | 3,817 (21.5) | <.001 | 2,819(12.60) | 2,318 (10.4) | <.001 |
| **Alcohol use, n (%)** | 8,432 (47.4) | 7,497 (42.1) | <.001 | 7,390(33.04) | 6,476 (29.0) | <.001 |
| **Exercise, n (%)** | 7,694 (43.2) | 12,726 (71.5) | <.001 | 13,454(60.15) | 16,867 (75.4) | <.001 |

1. **Normotensive patients (n=27,637)**

| **Risk factors** | **Statin (-)** | | | **Statin (+)** | | |
| --- | --- | --- | --- | --- | --- | --- |
|  | **(n=15,835)** | | | **(n=11,802)** | | |
|  | **Baseline** | **Follow-up** | **P value** | **Baseline** | **Follow-up** | **P value** |
| **Age (years)** | 51.2±8.9 | 60.1±8.7 | <.001 | 55.9±7.2 | 60.5±7.2 | <.001 |
| **BMI (kg/m^2^)** | 24.3±2.8 | 24.1±2.9 | <.001 | 24.2±2.7 | 24.2±2.8 | 0.969 |
| **SBP (mmHg)** | 125.5±16.2 | 125.8±15.2 | 0.067 | 123.9±14.8 | 123.0±13.9 | <.001 |
| **DBP (mmHg)** | 79.5±11.0 | 78.0±10.1 | <.001 | 77.4±10.1 | 75.9±9.2 | <.001 |
| **Total cholesterol (mg/dL)** | 258.6±22.9 | 227.1±34.5 | <.001 | 271.8±27.8 | 221.2±45.9 | <.001 |
| **FSG (mg/dL)** | 92.6±13.1 | 99.5±21.5 | <.001 | 94.0±11.6 | 98.1±18.6 | <.001 |
| **Smoking, n (%)** | 4,574 (28.9) | 3,573 (22.6) | <.001 | 1,584 (13.4) | 1,256 (10.6) | <.001 |
| **Alcohol use, n (%)** | 7,679 (48.5) | 6,878 (43.4) | <.001 | 3,818 (32.4) | 3,338 (28.3) | <.001 |
| **Exercise, n (%)** | 6,923 (43.7) | 11,503 (72.6) | <.001 | 7,233 (61.3) | 9,081 (76.9) | <.001 |

1. **Hypertensive patients (n=12,827)**

| **Risk factors** | **Statin (-)** | | | **Statin (+)** | | |
| --- | --- | --- | --- | --- | --- | --- |
|  | **(n=1,963)** | | | **(n=10,564)** | | |
|  | **Baseline** | **Follow-up** | **P value** | **Baseline** | **Follow-up** | **P value** |
| **Age (years)** | 59.6±9.9 | 67.5±9.5 | <.001 | 59.1±8.5 | 63.7±8.5 | <.001 |
| **BMI (kg/m^2^)** | 25.1±2.9 | 24.7±3.2 | <.001 | 25.1±2.9 | 25.0±3.0 | <.001 |
| **SBP (mmHg)** | 142.7±20.6 | 134.0±17.3 | <.001 | 135.9±17.3 | 130.2±14.6 | <.001 |
| **DBP (mmHg)** | 88.1±13.0 | 81.0±10.7 | <.001 | 83.9±11.0 | 79.2±9.4 | <.001 |
| **Total cholesterol (mg/dL)** | 259.6±27.0 | 221.1±36.4 | <.001 | 267.3±25.5 | 205.3±44.2 | <.001 |
| **FSG (mg/dL)** | 94.6±13.3 | 101.6±22.2 | <.001 | 96.1±12.1 | 101.0±18.6 | <.001 |
| **Smoking, n (%)** | 342 (17.4) | 244 (12.4) | <.001 | 1,235 (11.7) | 1,062 (10.1) | <.001 |
| **Alcohol use, n (%)** | 753 (38.4) | 619 (31.5) | <.001 | 3,572 (33.8) | 3,138 (29.7) | <.001 |
| **Exercise, n (%)** | 771 (39.3) | 1,223 (62.3) | <.001 | 6,221 (58.9) | 7,786 (73.7) | <.001 |

BMI, body mass index; DBP, diastolic blood pressure; FSG, fasting serum glucose; SBP, systolic blood pressure
