# Supplementary material for: Risk of new-onset diabetes among patients treated with statins according to hypertension and gender: Results from a nationwide health-screening cohort
Source: PLoS One. 2018 Apr 9;13(4):e0195459. doi: 10.1371/journal.pone.0195459 (PMC5891021; doi:10.1371/journal.pone.0195459)
Supplement: S2 Table — (DOCX) [file pone.0195459.s002.docx]

**S2 Table. Multivariate Cox regression analysis for development of new-onset type 2 diabetes mellitus according to sex and hypertension**

| Risk factors | Male | | | | Female | | | |
| --- | --- | --- | --- | --- | --- | --- | --- | --- |
|  | **HTN (-)** | | **HTN (+)** | | **HTN (-)** | | **HTN (+)** | |
|  | **(n=13,948)** | | **(n=4,934)** | | **(n=13,948)** | | **(n=4,934)** | |
|  | **HR**  **(95% CI)** | **P value** | **HR**  **(95% CI)** | **P value** | **HR**  **(95% CI)** | **P value** | **HR**  **(95% CI)** | **P value** |
| Statin use | 1.61  (1.35, 1.92) | <.001 | 1.18  (0.88, 1.60) | 0.274 | 1.76  (1.48, 2.10) | <.001 | 1.32  (1.03, 1.70) | 0.030 |
| Age | 1.04  (1.03, 1.05) | <.001 | 1.02  (1.00, 1.03) | 0.107 | 1.03  (1.02, 1.04) | <.001 | 1.01  (1.00, 1.02) | 0.191 |
| BMI | 1.09  (1.06, 1.12) | <.001 | 1.06  (1.01, 1.10) | 0.011 | 1.09  (1.06, 1.11) | <.001 | 1.07  (1.05, 1.10) | <.001 |
| SBP | 1.01  (1.00, 1.02) | 0.002 | 1.00  (0.99, 1.01) | 0.662 | 1.01  (1.01, 1.02) | 0.002 | 1.00  (0.99, 1.01) | 0.932 |
| DBP | 0.99  (0.98, 1.00) | 0.044 | 0.99  (0.97, 1.00) | 0.127 | 1.00  (0.99, 1.02) | 0.484 | 1.00  (0.99, 1.01) | 0.639 |
| FSG | 1.02  (1.02, 1.03) | <.001 | 1.03  (1.03, 1.04) | <.001 | 1.05  (1.05, 1.05) | <.001 | 1.04  (1.04, 1.04) | <.001 |
| TC | 0.99  (0.99, 0.99) | <.001 | 1.00  (0.99, 1.00) | 0.001 | 1.00  (0.99, 1.00) | <.001 | 0.99  (0.99, 1.00) | <.001 |
| Smoking | 1.21  (1.02, 1.43) | 0.030 | 1.05  (0.83, 1.35) | 0.670 | 1.34  (0.88, 2.06) | 0.174 | 1.90  (1.23, 2.93) | 0.004 |
| Alcohol use | 0.99  (0.84, 1.17) | 0.906 | 0.87  (0.68, 1.12) | 0.281 | 0.66  (0.50, 0.87) | 0.003 | 0.86  (0.65, 1.14) | 0.294 |
| Exercise | 0.74  (0.63, 0.88) | <.001 | 0.93  (0.72, 1.22) | 0.605 | 0.97  (0.82, 1.15) | 0.706 | 0.84  (0.70, 1.00) | 0.044 |
| FHx of DM | 1.35  (1.11, 1.64) | 0.003 | 1.00  (0.69, 1.45) | 0.986 | 1.47  (1.20, 1.79) | <.001 | 1.29  (1.03, 1.63) | 0.029 |
| Drug use* | 0.73  (0.40, 1.35) | 0.317 | 0.92  (0.72, 1.18) | 0.513 | 1.20  (0.92, 1.55) | 0.176 | 0.98  (0.82, 1.14) | 0.802 |

BMI, body mass index; CI, confidence interval; DBP, diastolic blood pressure; FSG, fasting serum glucose; HTN, hypertension; SBP, systolic blood pressure; TC, total cholesterol

*Drug use: use of thiazide or beta-blocker
